# Cellular uptake and anti-tumor activity of gemcitabine conjugated with new amphiphilic cell penetrating peptides

**DOI:** 10.17179/excli2017-249

**Published:** 2017-05-09

**Authors:** Parvin Zakeri-Milani, Samad Mussa Farkhani, Ali Shirani, Samaneh Mohammadi, Javid Shahbazi Mojarrad, Jafar Akbari, Hadi Valizadeh

**Affiliations:** 1 Liver and Gastrointestinal Diseases Research Center and Faculty of Pharmacy, Tabriz University of Medical Sciences, Tabriz, Iran; 2 Research Center for Pharmaceutical Nanotechnology and Department of Medical Nanotechnology, Faculty of Advanced Medical Sciences, Tabriz University of Medical Sciences, Tabriz, Iran; 3 Student Research Committee, Tabriz University of Medical Sciences, Tabriz, Iran; 4 Drug Applied Research Center and Faculty of Pharmacy, Tabriz University of Medical Sciences, Tabriz, Iran; 5 Department of Pharmaceutics, Faculty of Pharmacy, Mazandaran University of Medical Sciences, Sari, Iran

**Keywords:** cell penetrating peptide, gemcitabine, toxicity, drug delivery

## Abstract

Gemcitabine (Gem) is used as a single agent or in combination with other anticancer agents to treat many types of solid tumors. However, it has many limitations such as a short plasma half-life, dose-limiting toxicities and drug resistance. Cell-penetrating peptides (CPPs) are short peptides which may deliver a large variety of cargo molecules into the cancerous cells. The current study was designed to evaluate the antiproliferative activity of gemcitabine chemically conjugated to CPPs. The peptides were synthesized using solid phase synthesis procedure. The uptake efficiency of CPPs into cells was examined by flow cytometry and fluorescent microscopy. The synthesized peptides were chemically conjugated to Gem and the *in vitro* cytotoxicity of conjugates was tested by MTT assay on A594 cell line. According to the obtained results, cellular uptake was increased with increasing the concentration of CPPs. On the other hand the coupling of Gem with peptides containing block sequence of arginine (R5W3R4) and some alternating sequences (i.e. [RW]6 and [RW]3) exhibited improved antitumor activity of the drug. The findings in this study support the advantages of using cell-penetrating peptides for improving intracellular delivery of Gem into tumor as well as its activity.

## Introduction

Gemcitabine (Gem), a deoxycytidine analog, is used in the treatment of many types of solid tumors, including pancreatic, ovarian, breast, bladder, and lung cancers (Tao et al., 2012[41]; Martin-Banderas et al., 2013[27]). For activation this pro-drug requires cellular uptake and intracellular phosphorylation (Fujimura et al., 2014[17]). Inside the cell, gemcitabine is phosphorylated to gemcitabine monophosphate (dFdCMP) by deoxycytidine kinase (dCK), which is further phosphorylated to di- and tri-phosphorylated gemcitabine (dFdCDP and dFdCTP) (Derakhshandeh and Fathi, 2012[14]; Lansakara et al., 2012[22]). The metabolite, dFdCTP, is incorporated into DNA as a false nucleoside, eventually leading to inhibition of DNA polymerases, and induces cells to undergo apoptosis (Vandana and Sahoo, 2010[43]; Chung et al., 2012[9]). Although the molecular events mentioned above eventually contribute to the effectiveness of gemcitabine in fighting tumor cells, however the drug possesses certain demerits. Gemcitabine is converted into its inactive and more soluble metabolites, 2', 2'-difluorodeoxyuridine (dFdU) via deoxycytidine deaminase expressed in blood and various tumor tissues, causing a very short plasma half-life (Vandana and Sahoo, 2010[43]; Dalla Pozza et al., 2013[12]). Thus, a frequent administration schedule at high doses is required, leading to significant side effects such as hepatotoxicity and nephrotoxicity (Das et al., 2014[13]). Additionally, gemcitabine is too hydrophilic to passively cross the plasma membrane and its cellular uptake requires the presence of nucleoside transport proteins such as human equilibrative nucleoside transporter-1 (hENT1) (Bildstein et al., 2010[4]). Therefore, drug resistance associated with deficiencies in the expression of hENT1 confers lower gemcitabine toxicity in tumor cells by blocking the cellular transport of gemcitabine (Chung et al., 2012[9]; Tao et al., 2012[41]). 

Up to now various delivery strategies such as liposomes (Grazia Calvagno et al., 2007[18]; May et al., 2013[28]; Bersani et al., 2014[3]), nanoparticles (Lee et al., 2013[23]; Dolatabadi et al., 2015[15]), lipidic and nonlipidic derivatives (Bersani et al., 2014[3]), PEG and other polymeric drug conjugates (Aggarwal et al., 2013[1]; Chitkara et al., 2013[8]) have been investigated to prevent rapid plasma degradation and to improve delivery of gemcitabine to the tumor tissue. However, gemcitabine is a low molecular weight compound with high water solubility. At physiological pH it is mainly uncharged which may readily diffused out from the liposomal bilayers and nanoparticles resulting in low drug encapsulation efficiencies and rapid release rate (Chitkara et al., 2013[8]). Among the new strategies, the pro-drug approach has gained major interest which includes modifications on gemcitabine molecule and conjugation onto a lipoid or polymeric carrier (Chitkara et al., 2013[8]). However, the delivery of hydrophilic compounds by these carriers into cells is limited by their poor ability to penetrate through cell membrane. Therefore an alternative strategy, which has recently attracted much more attention, was established by chemically conjugating hydrophilic drugs to cell-penetrating peptides (CPPs).

PPs are short peptides which are able to deliver a large variety of cargo molecules into a wide range of cells and tissues in a nontoxic manner (Cohen-Avrahami et al., 2010[10]; Liu et al., 2013[25]; Sawant et al., 2013[37]; Copolovici et al., 2014[11]; Ren et al., 2014[35]). It has been demonstrated that conjugation of gemcitabine with polypeptide may improve its plasma stability and sustain the drug release profile (Kiew et al., 2010[21]). In recent years, numerous natural and synthetic CPPs, such as Tat, transportan, and polyamino acids (e.g., poly-arginines), were discovered or designed for intracellular delivery of anticancer agents (Gupta et al., 2011[19]; Nasrolahi Shirazi et al., 2013[31]). Among these CPPs, TAT peptide and poly-arginine are the most frequently used for the delivery of various molecules such as proteins, peptides, RNA, therapeutic and imaging compounds into cells (Shirazi et al., 2014[39]; Tsumuraya and Matsushita, 2014[42]). It has been shown that long poly-arginine generally results in more effective uptake, but the majority of studies suggest optimal lengths in the range of 8 to 15 arginines. Recently, it was revealed that the presence of tryptophan and backbone spacing can affect uptake efficiency as well as its mechanism (Rydberg et al., 2012[36]). Several small molecule chemotherapeutics, such as Taxol (Dubikovskaya et al., 2008[16]), methotrexate (Lindgren et al., 2006[24]), and doxorubicin (Nasrolahi Shirazi et al., 2013[32]) have shown improved activity when conjugated with CPPs.

The objective of this study was to examine the effect of gemcitabine coupling with arginine and tryptophan-rich CPPs on the toxicity of the drug in A549 cell line. Furthermore, intracellular uptake, and subcellular distribution of five synthesized CPPs were studied. 

## Materials and Methods

### Materials

Gemcitabine hydrochloride was purchased from Actavis (Italy). FMOC-Rink-Amide AM resin and amino acid derivatives were obtained from AAPPTec (Louisville, USA). Coupling agents (TBTU, DIEPA), scavengers (ethanedithiol, phenol, TIPS), cleavage reagents (piperidine, TFA), and FITC were purchased from Sigma (St. Louis, USA). 3-(4,5-dimethylthiazol-2-yl) -2,5-diphenyltetrazolium bromide (MTT), RPMI1640, fetal bovine serum (FBS), trypsin-EDTA and penicillin/streptomycin were purchased from Invitrogen (Carlsbad, USA). 

### Peptide synthesis

**Figure 1:**
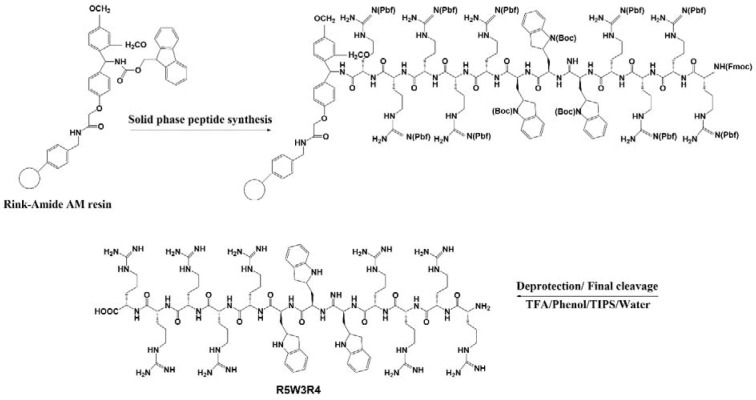
Schematic representing synthesis of R5W3R4 peptide

All peptides were synthesized manually by solid-phase peptide synthesis method on Rink-Amide AM resin by FMOC strategy in a fritted glass vessel (Mohammadi et al., 2015[30]; Shirani et al., 2015[38]). The resin was swelled in anhydrous DMF for about 1 h under dry nitrogen. FMOC deprotection of resin was carried out using piperidine in DMF (20 % v/v, 2 ml, 30 min). FMOC-Arg (Pbf) -OH (0.14 mmol) was coupled to the resin in the presence of TBTU (0.12 mmol) and DIPEA (50 μL) in DMF (2 ml) by mixing for 2 h. After completion of the coupling, the reaction solution was filtered off and the resin was washed with DMF (4 × 2 ml) and DCM (4 × 2 ml), followed by FMOC deprotection using piperidine in DMF. The resin was washed with DMF and DCM. Ninhydrin test was used to monitor FMOC deprotection and coupling of amino acids in each step. After the coupling of all amino acids, the resin was washed with DMF, DCM and ethanol (each 2 × 2 ml). The resin was dried under vacuum for 24 h. Fresh cleavage cocktail, reagent B, TFA/ TIPS / phenol /water (88:2:5:5 v/v/v/v, 3 ml), was added to the resin for side-chain deprotection and the final cleavage of the synthesized peptide from the solid support. The mixture was shaken at room temperature for 2 h. The resin was collected by filtration and washed with another 2 ml of fresh cleavage cocktail. Combined filtrates were evaporated to reduce the volume under dry nitrogen. The crude peptide was precipitated by adding 100 ml of diethyl ether and centrifuged at 4000 RPM for 5 min to obtain the solid precipitates. The obtained peptide was further washed with ether (2 ×50 ml) for 2 times and lyophilized (Table 1(Tab. 1), Figure 1(Fig. 1)).

### FITC labeling of peptides

N-terminal FMOC deprotection of each prepared peptide was carried out using piperidine in DMF. A solution of 1.1 equivalent of FITC in pyridine/DMF/DCM was prepared and added to the peptide-resin then mixed overnight. The completion of the reaction was checked using ninhydrin test. The resin was washed and final cleavage of the CPP-FITC conjugates from the resin was carried out according to the mentioned protocol. 

### Preparation of drug-peptide conjugates 

**Figure 2:**
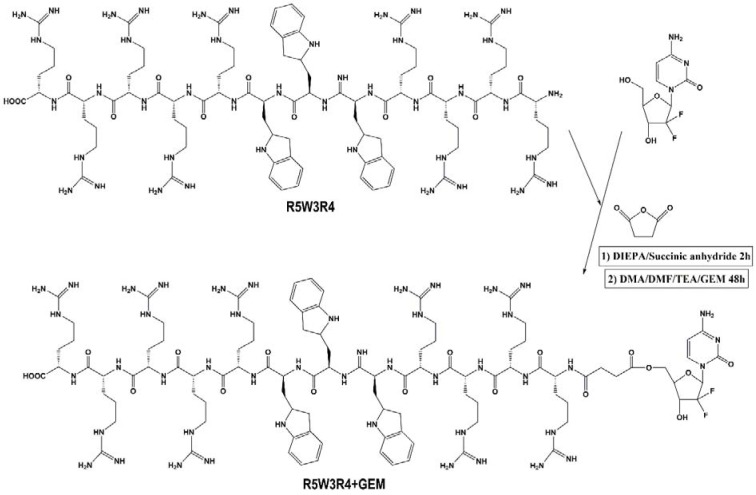
Schematic representing preparing of gemcitabine-R5W3R4 conjugate

Gemcitabine was coupled to peptides using a succinyl spacer by linking the amine group of the peptide to the hydroxyl group of the drug. The NH_2_ group of the peptide was changed to a carboxyl moiety by reaction with succinic anhydride as a linker. After synthesis of peptides on the resin, deprotected peptides were treated with succinic anhydride (1.5 eq.) and DIEPA (3 eq) in DMF for 2 h. The completion of the reaction was controlled by ninhydrin test. The resin was washed with DMF (4 × 2 ml) and DCM (4 × 2 ml). For conjugation of peptides to gemcitabine, TEA (50 µl) and DIEPA (3 eq) were added to a solution of 40 mg of gemcitabine in 3 ml of 85:15 (v/v) DMA/DMF mixture and was added to the succinylated peptide. The reaction mixture was kept under gentle stirring for 48 h. The mixture was then dried and drug-peptide conjugates were cleaved from the resin with TFA/ TIPS / phenol /water (88:2:5:5 v/v/v/v, 3 ml) cocktail (Figure 2(Fig. 2)).

### Cell culture

A549 lung carcinoma cell line was obtained from the Pasteur Institute (Iran). Cells were maintained in RPMI 1640 medium supplemented with 10 % FBS, 100 units/ml penicillin, and 100 μg/ml streptomycin and grown at 37 °C in a 5 % CO_2_ humidified atmosphere.

### In vitro cytotoxicity 

A549 cells were seeded into 96-well plates at a density of 1.5 × 10^4^ cells/well and pre-incubated for 24 h. The next day, different concentrations of peptides were added to the culture medium. Cells were incubated at 37 °C for 72 h. The medium was removed and the wells were washed with PBS. The MTT assay was performed by introducing 50 μl of 2 mg/ml MTT to each well for 4 h. The culture media aspirated and then resulting formazan crystals were dissolved in DMSO and the absorbance of individual wells was obtained at 570 nm. Untreated cells were defined as 100 % viable.

### Fluorescent microscopy 

The cellular uptake of the FITC-labeled peptide was examined in A549 cell line. A549 cells were seeded with RPMI 1640 medium on coverslips in 6-well plates and allowed to adhere overnight. Then the medium was removed and washed with PBS. The cells were treated with FITC-labeled peptide for 1.5 h at 37 °C. After incubation, the media containing the compound were removed and the cells were washed with PBS three times. Coverslips were placed on a microscope slide (Olympus IX81 fluorescence microscope, Olympus Optical Co., Ltd., Tokyo, Japan).

### Flow cytometry

A549 cells were seeded with RMPI 1640 medium in 6-well plates (3×10^5 ^cells/well) 24 h prior to the experiment. After 24 h, the medium was removed and washed with PBS. Then FITC-labeled peptide was added to the cells. The plates were incubated for 1.5 h at 37 °C. After that, the medium containing the peptide was removed. The cells were washed three times with PBS and detached with 0.25 % trypsin/EDTA (0.53 mM) for 5 min. To each well, 2 ml of the medium was added and centrifuged for 4 min. Cells were washed twice with PBS and finally were re-suspended in flow cytometry buffer and analyzed by FACSCalibur (Becton Dickinson, San Jose, CA, USA).

## Results

### Synthesis of CPPs and preparing drug-CPP conjugates

FMOC solid-phase peptide synthesis on the Rink-Amide AM resin was used for synthesizing linear peptides R5W3R4, [RW]3, [RW]4, [RW]5, and [RW]6 with hydrophobic (W) and charged (R) residues. FITC was coupled to synthesized peptides to investigate their uptake efficiency. Drug-CPP conjugates were prepared by using succinic linker. UV-Vis spectroscopy was used for controlling the drug loading to peptides. 

### Cellular toxicity of the CPPs

**Figure 3:**
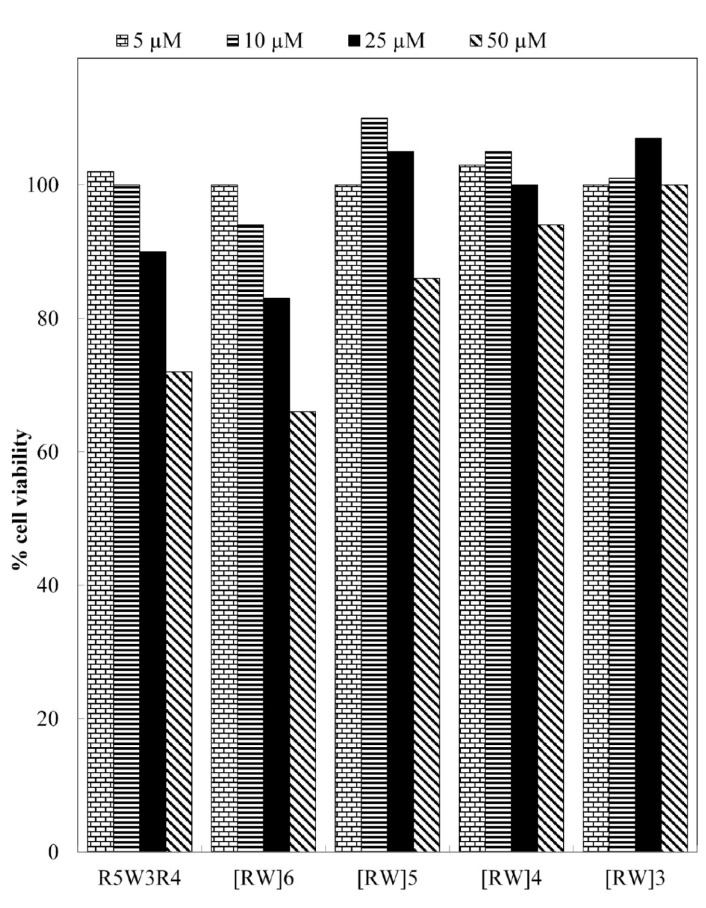
Cytotoxicity of peptides on A549 cells after 72 h incubation

As a functional delivery vector of anti-cancer drugs, blank CPPs must have high uptake efficiency with low levels of toxicity against cells (Soler et al., 2014[40]). Therefore, all peptides were examined for their toxicity in A549 cells before examining the cellular toxicity of drug-CPP conjugates. The cytotoxicity was determined by MTT assay after 72 h of peptide exposure in concentration range between 5 to 50 µM. As shown in Figure 3(Fig. 3), the peptides exhibited no toxicity up to the concentration of 10 µM. Among five CPPs, [RW]4 and [RW]3 did not show toxicity even at 50 µM. However, R5W3R4 and [RW]6 exhibited cell toxicity value of 9 % and 16 % at the concentration of 25 µM, respectively. At the concentration of 50 μM, cell death caused by R5W3R4, [RW]6, and [RW]5 were 28 %, 34 % and 14 % respectively. Thus, because of low toxicity, concentration of 25 μM was selected for cell-based studies of drug-CPP conjugate. 

### Cellular uptake 

**Figure 4:**
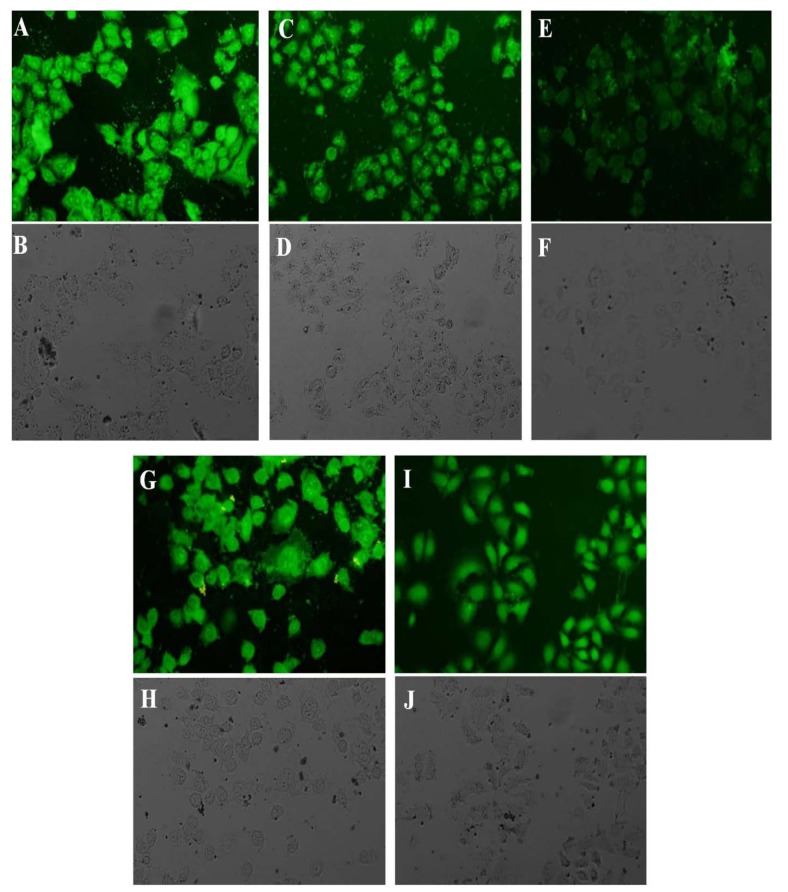
Fluorescence microscopy, visualization of FITC-labeled, R5W3R4 (A, B), [RW]6 (C, D), [RW]5 (E, F), [RW]4 (G, H), and [RW]3 (I, J) in A459 cells. The top photos show fluorescence microscopy and the bottom bright field of A459 cells. Live cells were treated with 25 µm of peptides for 1.5 h at 37 °C. Control cells were incubated in RPMI medium without the peptides.

The uptake and intracellular localization of FITC-labeled peptides were examined by fluorescent microscopy. Figure 4(Fig. 4) shows the intracellular distribution of the prepared peptides following 1.5 h incubation. The uptake of all peptides was examined at the concentration of 25 µM. [RW]5 exhibited the lowest uptake into the cells among five peptides. After entering the cell, much of [RW]5 accumulated around the nucleus. R5W3R4, [RW]6 and [RW]4 displayed homogeneous staining throughout the intracellular space. However, stronger intensity was observed in cellular structures that are morphologically identified as the cell nucleus and nucleoli. Fluorescent image revealed that [RW]3 enters mainly into the cell nucleus. 

**Figure 5:**
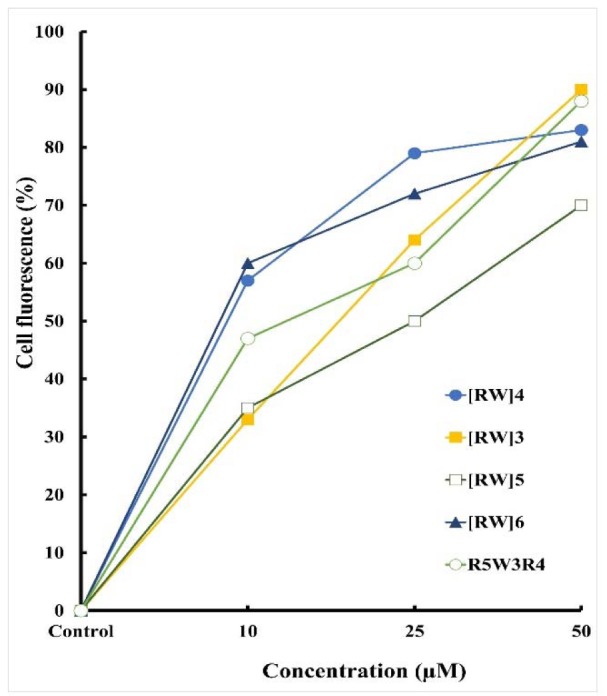
Cellular uptake of FITC-labeled peptides in live A549 cells after incubation. The uptake was measured as the relative fraction of positive cells (%) from flow cytometry analysis of all living cells positive for the fluorophore.

Flow cytometry was used to determine the relative amounts of internalized peptide after 1.5 h incubation at 37 °C. After the incubation, cells were treated with trypsin to remove the cell surface-bound peptides. Figure 5(Fig. 5) shows the relative fraction of positive cells (%) after treatment with peptides. The percentage of cell fluorescence was increased with increasing peptides concentration. This effect was nearly linear for the tested concentrations. There was an increase in fluorescence intensity of cells treated with peptides of higher amino acid content. However, the [RW]5 exhibited lower levels of intracellular fluorescence. R5W3R4 and [RW]3 with three tryptophan showed maximum intracellular fluorescence relative to other peptides. 

### Antitumor performance of drug loaded CPPs

**Figure 6:**
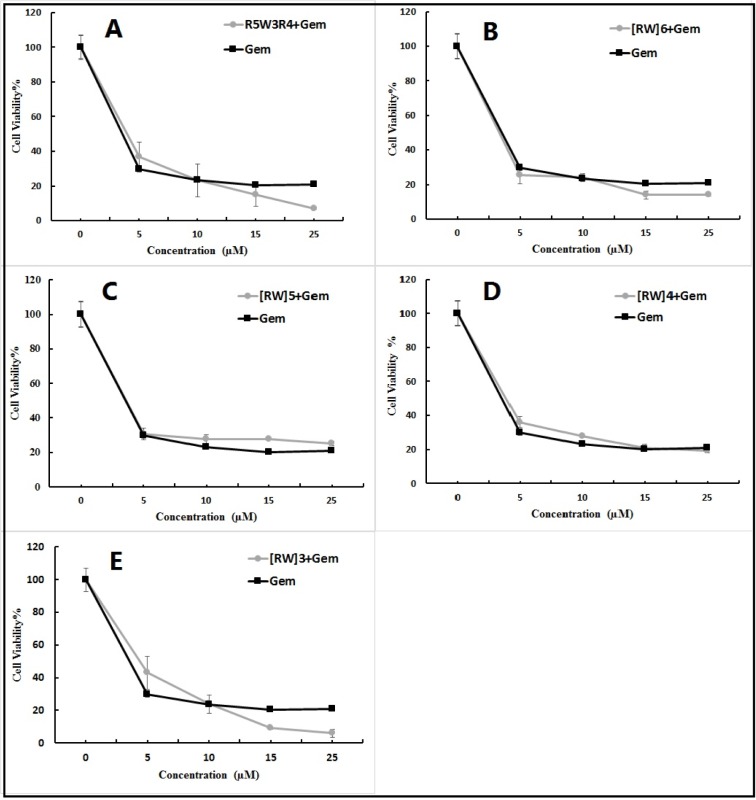
Toxicity of Gem-R5W3R4 (A), Gem-[RW]6 (B), Gem-[RW5] (C), Gem-[RW]4 (D), Gem-[RW]3 (E) and Gem to A549 cells. The cells were incubated for 3 days in 10 % FBS with or without peptides and analyzed for proliferation by MTT assay.

The antitumor activity of free gemcitabine and its conjugates with CPPs (Gem-CPP) was investigated using MTT test after incubation for 72 h. The activity of drug-peptide conjugates i.e. Gem-R5W3R4, Gem-[RW]6, Gem-[RW]5, Gem-[RW]4 and Gem-[WR]3, was evaluated and compared with that of the free drug (Figure 6(Fig. 6)). Drug loaded CPPs at concentrations less than 10 µM did not exhibit increased anti-proliferative activity compared to the free drug. However, at 15 and 25 µM, Gem-R5W3R4, Gem-[RW]6 and Gem-[WR]3 exhibited decreased cell viability. Free drug showed 20 % cell viability at concentrations of 15 and 25 µM. The cell viability value was reduced to 16 % and 6 % with Gem-R5W3R4 at 15 and 25 µM, respectively. In the case of Gem-[RW]6, cell viability was decreased to 14 % at 15 and 25 µM. Among the five peptide-drug conjugates, Gem-[RW]3 displayed the highest cytotoxicity at 15 and 25 µM. The cell viability of the drug was decreased to 9 % and 5 % when it was coupled to [RW]3 at 15 and 25 µM respectively. In the case of Gem-[RW]5 and Gem-[RW]4 conjugates, cell viability was slightly increased in comparison to the free Gem drug. 

The enhanced cell toxicity of drug-peptide conjugates could be attributed to the high cellular uptake tendency of the prepared peptides (Aroui et al., 2010[2]) demonstrated by the flow cytometry and fluorescent microscopy studies (Figures 4(Fig. 4) and 5(Fig. 5)). 

## Discussion

Gemcitabine has a therapeutic activity against a variety of solid tumors (Chung et al., 2012[9]; Zhao et al., 2012[44]). However, this anticancer drug suffers from serious limitations. Gemcitabine has very short plasma circulation time and high hydrophilicity, resulting in limited intracellular diffusion. In addition, cancer cells acquire resistance over time, which becomes a major concern for most gemcitabine-related chemotherapies (Maksimenko et al., 2013[26]). The resistance of tumor is related to the mechanism of internalization of this drug. Transport of gemcitabine into the cell requires both the concentrative (hCNT) and equilibrative (hENT) nucleoside transporters. Considering that cellular uptake of gemcitabine is mainly mediated by hENT1 transporters, hENT1-deficient cells and decreased expression of hENT1 is accounted for decreased gemcitabine toxicity by blocking the cellular uptake of the drug (Pili et al., 2010[34]). Coupling of anticancer drug to CPP may result in numerous advantages, such as improved solubility, intracellular uptake, bio-distribution and pharmacokinetic profiles. CPP-based drug delivery system offers great potential for improving intracellular delivery of therapeutic agents with poor permeability (Aroui et al., 2010[2]; Cohen-Avrahami et al., 2010[10]; Huang et al., 2013[20]; Liu et al., 2013[25]; Ren et al., 2014[35]). In this study, in order to protect gemcitabine from rapid metabolic inactivation and to improve its cell penetration, some pro-drugs were designed by coupling gemcitabine to CPP. This strategy could be used in fighting hENT1-deficient and resistant tumor cells by increasing transport of the gemcitabine into the cells.

After synthesis of peptides, the uptake efficiency was investigated. Then, gemcitabine was covalently attached to the peptides by using succinyl hydrolysable spacer which allows for the drug release after uptake into the cells (Cavallaro et al., 2006[7]). The cytotoxic efficacy of the free drug and drug-CPP conjugates was evaluated. The peptide sequences were chosen to examine how the presence of tryptophan and its position within the poly-arginine may influence the cellular uptake and cytotoxicity of the drug. It was evident that the addition of tryptophan to oligo-arginine could increase cellular uptake efficiency. Peptides with tryptophans in the middle, or evenly distributed along the peptide sequence exhibited higher uptake. This observation was in consistency with earlier reports (Rydberg et al., 2012[36]). By increasing the number of amino acids in the sequences the toxicity was improved so that Gem-R5W3R4 and Gem-[RW]6 conjugates with 12 residues exhibited the highest toxicity in cancer cells. 

The results showed that three of five peptides improved cytotoxicity of gemcitabine. Gem-R5W3R4, Gem-[RW]6 and Gem-[RW]3 conjugates displayed increased toxicity compared to free Gem. The increased toxicity of these drug-CPP conjugates was seen at 15 and 25 µm. One of the possible reasons for this effect might be the mechanism of CPP cellular uptake. Recent studies showed that endocytic pathways are the major route for internalization of CPPs. Although the endocytosis pathway may be responsible for the vast majority of cationic peptide internalization, numerous evidences suggest that direct penetration does occur at threshold concentrations (Palm-Apergi et al., 2012[33]). It was shown that at low concentration, endocytosis of peptides could occur which may result in endosomal entrapped peptides and possible metabolic degradation (Mellert et al., 2012[29]; Brock, 2014[5]). However at higher concentrations (above 10 µm), direct translocation into the cell is predominant. With the direct uptake, the drug molecules delivered by CPP would not fall into the endosome. Possibly, direct uptake of drug-CPP conjugates at higher concentrations is one of the reasons for increased toxicity of gemcitabine. In addition to improved cytoplasmic delivery, it may also represent a valuable strategy to overcome drug resistance. The main mechanisms recognized for multidrug resistance is the presence of P-glycoprotein in the plasma membrane, which can extrude a wide range of anticancer drugs. The ability of CPP-drug conjugates to evade the P-gp efflux pump was confirmed using several assays (Castex et al., 2004[6]; Aroui et al., 2010[2]). This leads to higher intracellular drug concentration causing higher toxicity in resistant cell lines.

In conclusion, the obtained results showed that the coupling of gemcitabine to CPPs including R5W3R4, [RW]6 and [RW]3 may cause the increased antitumor activity of the drug. Collectively, the findings in this study support the advantages of using CPPs for improving intracellular delivery of drugs into tumor cells as well as their activity. Furthermore, it is possible to overcome gemcitabine resistance associated with deficiencies in the expression of hENT1 by using CPP strategy. In the future work the *in vivo* effect of the gemcitabine-CPP conjugates will be evaluated. Furthermore the investigation of the effect of CPPs on the other pharmacokinetic parameters of the drug is worthy.

## Acknowledgement

The financial support from the "Drug Applied Research Center" and "Research Center for Pharmaceutical Nanotechnology" of Tabriz University of Medical Sciences is greatly acknowledged. This paper is based on an MSc thesis submitted by S.M. Farkhani in Faculty of Advanced Medical Sciences, Tabriz University of Medical Sciences.

## Conflict of interest

The authors report no conflicts of interest.

## Figures and Tables

**Table 1:**
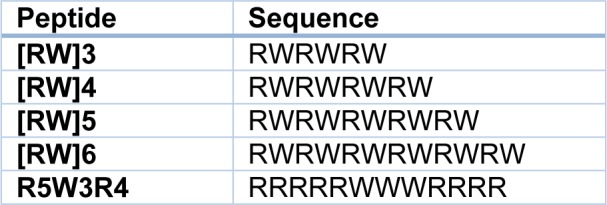
Peptide sequences
